# Degradation of Polycyclic Aromatic Hydrocarbons by Moderately Halophilic Bacteria from Luzon Salt Beds

**DOI:** 10.5696/2156-9614-8.19.180915

**Published:** 2018-09-10

**Authors:** Carolyn L. Nanca, Kimberly D. Neri, Anna Christina R. Ngo, Reuel M. Bennett, Gina R. Dedeles

**Affiliations:** 1 The Graduate School, University of Santo Tomas, Manila, Philippines; 2 Laboratory of Pure and Applied Microbiology, Research Center for the Natural and Applied Sciences, University of Santo Tomas, Manila, Philippines; 3 Collection of Microbial Strains, University of Santo Tomas, Manila, Philippines; 4 Department of Biological Sciences, College of Science, University of Santo Tomas, Manila, Philippines; 5 Department of Sciences, Senior High School, University of Santo Tomas, Manila, Philippines

**Keywords:** halophiles, salt bed, polycyclic aromatic hydrocarbons, biodegradation, pyrene, fluorene, fluoranthene

## Abstract

**Background.:**

Polycyclic aromatic hydrocarbons (PAHs) are common environmental contaminants which are highly toxic due to their carcinogenic and mutagenic effects. They are released into the environment by incomplete combustion of solid and liquid fuels, accidental spillage of oils and seepage from industrial activities. One of the promising processes mitigating PAHs is through biodegradation. However, conventional microbiological treatment processes do not function well at high salt concentrations. Hence, utilization of halophilic bacteria should be considered.

**Objectives.:**

This study aimed to assess the ability of halophilic bacteria isolated from local salt beds in Pangasinan and Cavite, the Philippines, to degrade PAHs pyrene, fluorene and fluoranthene.

**Methods.:**

Polycyclic aromatic hydrocarbon-tolerant halophilic bacteria collected from two sampling sites were phenotypically characterized, molecularly identified and tested to determine their potential to degrade the PAHs pyrene, fluorene and fluoranthene at a hypersaline condition. Best PAH degraders were then assayed to identify the optimal degradation using such parameters as pH, temperature and PAH concentration. Testing for enzyme degradation was also done to determine their baseline information. Extraction and analysis of degraded PAHs were performed using centrifugation and UV-vis spectrophotometry.

**Results.:**

Twelve isolates from both collection sites tolerated and grew in culture with selected PAHs. These were identified into four genera (Halobacillus, Halomonas, Chromohalobacter, and Pontibacillus). Selected best isolates in a series of biodegradation assays with the above-mentioned parameters were Halobacillus B (Collection of Microbial Strains (CMS) 1802) (=trueperi) (Gram-positive) for pyrene and fluoranthene, and Halomonas A (CMS 1901) (Gram-negative) for fluorene. Degrader biomass and PAH degradation were invariably negatively correlated. Qualitative tests with and without peptone as a nitrogen source implied enzymatic degradation.

**Discussion.:**

Polycyclic aromatic hydrocarbons utilized by these halophilic bacteria served as a sole source of carbon and energy. Implications of biodegradation of the two best isolates show that high molecular weight (HMW) (4-ring) pyrene tends to be degraded better by Gram-positive bacteria and low molecular weight (3-ring) fluorene by Gram-negative degraders.

**Conclusions.:**

Halophilic bacteria constitute an untapped natural resource for biotechnology in the Philippines. The present study demonstrated their potential use in bioremediation of recalcitrant hydrocarbons in the environment.

**Competing Interests.:**

The authors declare no competing financial interests.

## Introduction

Polycyclic aromatic hydrocarbons (PAHs) are composed of two or more fused aromatic rings.^1^ These macrocyclic compounds are ubiquitous environmental pollutants of both natural and anthropogenic origin, coexisting generally in areas of oil fields and gas factories. Air, soil, water and vegetation all act as environmental sinks for PAHs. Their widespread occurrence in the environment, level of recalcitrance to biodegradation, bioaccumulation potential and carcinogenic activity pose a serious concern to the health of aquatic life and humans.^2^

The concern for PAHs parallels that for its most common natural source - petroleum oil, an abundant and well-known hydrocarbon which ranks worldwide as the most valuable traded commodity for use to generate energy needed in industry.^3^ In the Philippines, transportation of crude oil and petroleum by oil tankers is a lifeline, as the country depends largely on imports for its energy needs. A single accident may result in the spill of crude oil or petroleum products that can cause massive damage to the marine environment and nearby-to-distant coastal areas, affecting human and animal life. The hazards associated with PAHs and similar hydrocarbons require effective remediation strategy. However, conventional microbiological treatment processes do not function well at a high salt concentration.^4^ Several studies suggest that halophilic bacteria may have greater potential to degrade hydrocarbons, PAHs included, especially in marine sediments.^4–7^

Halophilic bacteria and archaea are unique prokaryotes able to withstand very high salt concentrations known in hypersaline environments. Vast areas on the planet are hypersaline, exposed to contamination with hydrocarbons, but are among the least studied extreme environments.^8–10^ Hypersalinity is typical of salterns, sites with a salt content 5–10 times higher than in seawater (150–300 g/L) and which undoubtedly also harbor halophiles.^11^ In Southeast Asia, particularly in the Philippines, however, knowledge of the diversity of halophilic microorganisms and their capacity to degrade hydrocarbons is very limited.^12^

In the present study, halophilic bacteria indigenous in local salt beds were explored for their ability to degrade the PAHs pyrene, fluorene and fluoranthene. Emphasis was given to fundamental growth parameters, including pH, temperature and salinity to provide baseline information on the culture of the halophiles and their degradation of selected PAHs under a saline condition. Results were aimed at future biotechnological application of the isolates singly or in combination in oil pollution mitigation.

## Methods

Marine Agar and Marine Broth 2216 (HiMedia, India) supplemented with 0.15% sodium chloride (NaCl) were used as the culture media for the isolation and subsequent biodegradation assays of PAHs of halophilic bacteria isolated from salt beds. The PAHs pyrene, fluorene, and fluoranthene with 98% purity and N,N-dimethylformamide (DMF) were purchased from Sigma-Aldrich (USA). Reagents, all of analytical grade, were obtained from RCI Labscan, Thailand.

Stock solutions of pyrene, fluorene, and fluoranthene, the model pollutant PAHs, were prepared following the procedure of Bezalel *et al*.^13^ Then pyrene and fluorene at 2.5 mg were added to 0.3 mL DMF each for a final concentration of 8.33 mg/mL. For fluoranthene, 20 mg was dissolved in 0.5 mL of DMF for an equivalent concentration of 40 mg/mL. Prepared stock solutions (30 mL) were kept in the dark to avoid photooxidation.^14^

### Collection of salt and soil samples

Thirty-five salt sediments and soil samples were collected. Ten salt samples and eight and seven soil samples were obtained, respectively, from two local salt beds in Dasol, Pangasinan and Noveleta, Cavite, the Philippines. One gram of each sample was enriched in 1 L of seawater supplemented with 5-g peptone and 1-g yeast extract. Separate triplicate aliquots from each 3-day-old enrichment were plated on Marine Agar with 0.15% NaCl following the procedure of Yoon *et al*.^15^ Cultures were incubated at room temperature for 5–7 days and, thereafter, resulting isolated colonies were selected and purified by re-streaking on Marine Agar.

**Abbreviations**
*DMF*: N,N-dimethylformamide
*HMW*: High molecular weight
*LMW*: Low molecular weight

### Identification of PAH-tolerant halophilic bacteria

Marine Agar plates with 0.15% (w/v) NaCl and individually supplemented with 1% of the PAH stock solutions were streaked with the selected isolates incubated at room temperature for 5–7 days and then observed for the presence or absence of growth.^16^ Isolates able to grow in Marine Agar plates with PAH were morphologically characterized with emphasis on Gram staining reaction, cell shape and size, and colony color, shape, size, surface, edge, elevation and opacity. Biochemical tests for catalase, oxidase, motility, indole, urease, sugar fermentation, H2S production and for hydrolysis of casein, gelatin, starch and Tween 80 were also done for further characterization of the isolates.^6^,^17–20^ The media used for all biochemical tests were also supplemented with NaCl to support the growth of the halophilic bacteria.^19^ Results were analyzed using Bergey's Manual of Systematic Bacteriology, Second Edition and compared to minimal standards from related journals to partially identify the isolates up to at least the generic level.^21^

For identification at the species level, molecular analysis (16S) was performed with the use of Wizard^®^ Genomic DNA Purification Kit (Promega), following the manufacturer's instructions. Amplification of the 16S rRNA region of the isolates by polymerase chain reaction (PCR) was performed with primers 27F (5'-AGA GTT TGA TCM TGG CTC AG-3') and 1492R (5'-TAC GGY TAC CTT GTT ACG ACT T-3'). The purified DNA samples and the primers were sent to Macrogen (Seoul, Korea) for sequencing. Sequences of isolated halophilic bacteria and type strains obtained from NCBI GenBank were uploaded to the TrEase webserver for multiple sequence alignment using multiple alignment using fast Fourier transform (MAFFT).^22^ A neighbor joining tree was generated using the MEGA software version 7.0 following the Tamura-Nei model at 1000 bootstrap replications.^23^

### Biodegradation assay of PAH-tolerant halophilic bacteria

The biodegradation potential of isolates under hypersaline conditions was determined using a modified protocol of Bezalel *et al*.^13^ The experimental setup for biodegradation was Marine Broth 2216 with 0.15% NaCl (9.8 mL), standardized bacterial inocula (0.1 mL), and 0.1 mL each of the stock solutions of the test PAHs in DMF. Fresh enriched bacterial isolates were standardized (McFarland 0.5 = 1.5 × 10^8^ CFU/mL) and inoculated into flasks containing the hydrocarbons. For the control, bacterial isolates were standardized similarly in 0.15% saline solution. The setup was prepared in triplicates and kept in the dark. One flask without bacteria served as the negative control for the determination of any volatilization of hydrocarbon. Another flask with bacteria but no hydrocarbon served as the positive control. Rate of degradation was monitored on the 1st, 3rd, 5th, and 7th day of incubation as the standard procedure. Bacterial growth based on an aliquot of the setup was measured spectrophotometrically at OD_600_ nm.^24^

### Culture optimization for degradation by the best degraders

The isolates that exhibited the best degradation potential were tested again, this time with the parameters of pH, temperature and PAH concentration. Modifying the method of Zhou *et al.*, three triplicate sets of flasks with Marine Broth supplemented with the optimum salt concentration were prepared: the first set at pH 5.0, 7.0 and 9.0 at room temperature (25°C), the second at 25, 30, and 40°C, and the third at 1, 3 and 5% of the PAHs, also at room temperature.^16^ All culture flasks were incubated for 7 days with constant shaking. The rates of degradation as well as the growth of bacteria were analyzed as described above.

The isolates that passed co-culture assays (antagonism assay) were then mixed in Marine Broth with 0.05% NaCl and 1% of stock solutions of PAHs. Triplicate flasks for each of the PAHs were inoculated with the mixed culture of the two isolates standardized to 0.5 McFarland. Incubation condition and rates of degradation were monitored using the same standard procedure.

### Microbial growth on modified medium

In order to provide baseline data regarding enzymatic degradation of PAHs by the isolates, a qualitative test was done using three sets of plain agar media (HiMedia, 1.5%) with 0.05% NaCl; set 1 with 5% peptic digest of animal tissue as a nitrogen source plus 0.10% each of the three PAHs, set 2 with the individual PAH, and set 3 with peptic digest only. Triplicate plates for each set were streaked with the isolates on test. All were incubated at room temperature for 5–7 days and observed thereafter for the presence or absence of growth.

### Extraction and analysis of PAH degradation

Adapting the protocol of Olmos-Espejel *et al.*, 2-mL aliquot of the suspension was placed in a sterile Falcon conical tube.^25^ The suspension was acidified to pH 2.0 using 6N hydrochloric acid, killing the viable cells. Liquid/liquid extraction of the suspension was performed by adding 4 mL ethyl acetate, followed by mixing in a vortex and centrifugation (Jouan C3i, France) at 5,000 rpm for 5 mins to remove the biomass and to separate the organic and aqueous layers. The organic layer was analyzed using a UV-vis spectrophotometer (Beckman Coulter DU 730) with absorbance at 297 nm for pyrene, 299 nm for fluorene, and 302 nm for fluoranthene. Wavelengths were determined using the wavelength scan program of the UV–vis spectrophotometer. Ethyl acetate was used as the blank and the medium with PAH only was used as the standard.

Degradation of PAHs in percentage was computed using the formula of Yu *et al.*, shown in Equation 1.^26^

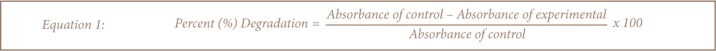



### Statistical analyses

To evaluate the rates of PAH degradation among the isolates, the mean values were subjected to two-way analysis of variance (ANOVA) at a 0.5% significance level, as well as a test for homogeneity and post hoc Tukey multiple comparison test using the Statistical Package for the Social Sciences (SPSS) software version 13.0 (SPSS, Inc., Chicago, IL, USA).^27^ For culture optimization, the data were analyzed using Taguchi methods with L9 orthogonal array as the experimental design.^28^

## Results

A combined total of 135 bacterial isolates were brought into axenic culture from the 35 salt and soil samples randomly collected from salt beds in Dasol, Pangasinan (DP) (18) and Noveleta, Cavite (NC) (17). Seventy-seven (77) isolates came from salt samples (DP = 41, NC = 36) and 58 from soil samples (DP = 33, NC = 25). All grew well in Marine Agar plates supplemented with 15% NaCl, hence, were halophilic. Twelve (9%) of the 135 halophiles were able to grow profusely in Marine Agar plates separately supplemented with pyrene, fluorene, and fluoranthene, indicating their potential to degrade PAHs by using PAHs as sole carbon sources. Relative to source materials, five and two of these isolates from DP and two and three from NC were isolated, respectively, from salt and soil samples. The bulk of excluded isolates (123 = 91%) from both types of sample was surprising. These isolates, although halophilic, individually could well be non-degraders of the PAHs used or, if otherwise, did not grow given the amount of PAHs used in the test on Marine Agar.

### Identification of PAH-tolerant halophilic bacteria

**Figure 1:**
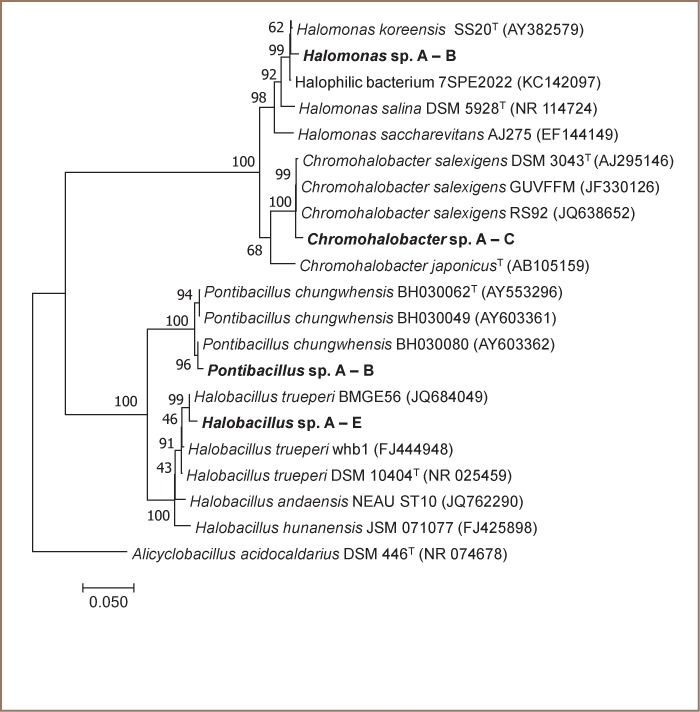
Neighbor joining tree for the PAH-degrading halophilic bacteria. Generated consensus sequences for Halomonas sp. A – B (DP – 17CY and NC– 15BY), Chromohalobacter sp. A – C (DP – 03APW, DP - 05CPW and NC – 07BPW), Pontibacillus sp. A – B (DP – 04BW and DP – 12CW), and Halobacillus sp. A – E (DP – 05BO, DP – 02APO, NC – 10BO, NC – 12APO and NC – 16CPO). Scale bar indicates number of nucleotide substitutions. Strain numbers and GenBank accession numbers are indicated after taxon names, respectively

Results of the identification by phenotypic means were in accordance with Bergey's Manual of Systematic Bacteriology, 2nd edition and other related journal articles containing minimal standards for identification to that level *(Table 1).*^21^ Of the 12 isolates which were all bacilli, seven from DP were of the genera Halobacillus, Chromohalobacter and Pontibacillus, each with two isolates, and Halomonas with one isolate. The other five isolates from NC consisted of Halobacillus with three isolates, and Halomonas and Chromohalobacter, each with one isolate. Among the four genera to which the combined DP and NC isolates belong, Halobacillus and Pontibacillus are Gram-positive, and Chromohalobacter and Halomonas, Gram-negative. Based on 16S rRNA analysis (*Figure 1*), Halomonas sp. A – B were conspecific to H. koreensis, Chromohalobacter sp. A – C to C. salexigens, Pontibacillus sp. A – B to P. chungwhensis, and Halobacillus sp. A – E to H. trueperi. All isolates were deposited in the University of Santo Tomas Collection of Microbial Strains and were given accession codes (Halobacillus spp. A–E [University of Santo Tomas, Collection of Microbial Strains (CMS) 1801 to 1805, respectively]; Halomonas sp. A–B [CMS 1901–1902]; Chromohalobacter sp. A–C [CMS 2201–2203] Pontibacillus chungwhensis A–B [CMS 2101–2102].

**Table 1:** Phenotypic and Genotypic Identities of PAH-tolerant Halophiles

**Initial Isolate Code**	**Morphological^+^**	**Biochemical^+^**	**16S rRNA**	**Final Isolate Code**
***DP-02APO***	Halobacillus	Halobacillus	Halobacillus trueperi	Halobacillus A
***DP-05BO***	Halobacillus	Halobacillus	Halobacillus trueperi	Halobacillus B
***NC-10BO***	Halobacillus	Halobacillus	Halobacillus trueperi	Halobacillus C
NC-12APO^**^	Halobacillus	Halobacillus	Halobacillus trueperi	Halobacillus D
NC-16CPO^* *^	Halobacillus	Halobacillus	Halobacillus trueperi	Halobacillus E
***DP-03APW***	Chromohalobacter	Chromohalobacter	Chromohalobacter salexigens	Chromohalobacter A
***DP-O5CPW***	Chromohalobacter	Chromohalobacter	Chromohalobacter salexigens	Chromohalobacter B
***NC-07BPW***	Chromohalobacter	Chromohalobacter	Chromohalobacter salexigens	Chromohalobacter C
DP-17CY ^**^	Halomonas	Halomonas	Halomonas koreensis	Halomonas A
NC-15BY ^**^	Halaomonas	Halomonas	Halomonas koreensis	Halomonas B
***DP-04BW***	Pontibacillus	Pontibacillus	Pontibacillus chungwhensis	Pontibacillus A
DP-12CW ^**^	Pontibacillus	Pontibacillus	Pontibacillus chungwhensis	Pontibacillus B

^**^ Isolates from soil samples; all others from salt samples

^+^ Bergey's Manual of Systematic Bacteriology 2nd ed (2005)^21^

**Abbreviations**: DP, isolates from Dasol, Pangasinan; NC, isolates from Noveleta, Cavite

### Biodegradation assay of PAH-tolerant halophilic bacteria

The 12 PAH-tolerant halophiles were tested individually for their comparative capacity to degrade PAHs in Marine Broth under a hypersaline condition. Degradation and biomass were monitored after 1, 3, 5, and 7 days of incubation in the dark at room temperature and with constant shaking.

**Figure 2:**
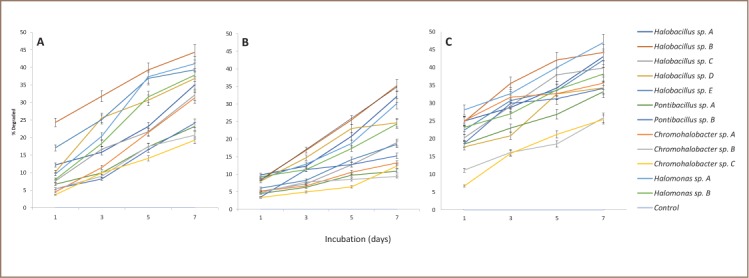
Biodegradation of (A) pyrene, (B) fluoranthene, and (C) fluorene after 7 days of incubation (N = 3)

Percent degradations of each PAH by the 12 isolates are shown in Figure 2. Parallel with the initial tests for tolerance to these hydrocarbons, the line graphs clearly demonstrate continuous significant degradation and, hence, utilization of the PAHs as carbon sources as a function of incubation time. Conversely, biomass decreased with incubation time. Degradation was faster with pyrene and fluoranthene than with fluorene, but it was higher with pyrene and fluorene than with fluoranthene. Figure 2 also indicates that degradation differed significantly among isolates irrespective of generic affiliation and likewise between or among isolates within a genus; where Halobacillus B and Halomonas A were the top degraders.

As expected, degradations of the PAHs by the top two degraders were inversely related to biomass, i.e. degradation decreased as biomass increased or vice versa. For the two 4-ring isomers pyrene and fluoranthene, degradation by the same isolate Halobacillus B was faster and higher with the former than with the latter PAH. Corresponding degradation in percentage after day 7 was higher by 9% for pyrene (44%) over fluoranthene (35%).

**Figure 3:**
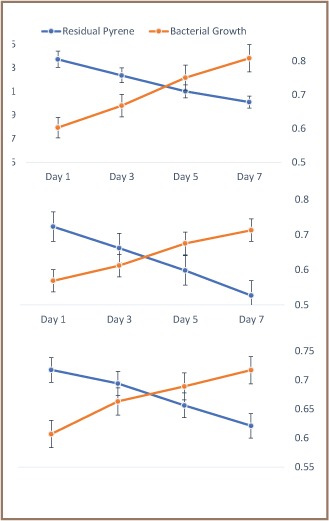
Time-course degradation of (A) pyrene, (B) fluoranthene by Halobacillus sp. B, and (C) fluorene by Halomonas A (N = 3)

Rates of degradations of the PAHs arrived at in the present study were up to less than 50% (fluorene = 47%, pyrene = 44%, fluoranthene = 35%) (Figures 2 and 3a–c), and biomass buildup by all the degraders, the top two included, was up to log phase only. To what extent both degradation parameters would have gone had incubation time been extended beyond 7 days is worth further investigation.

### Culture optimization for degradation by the best degraders

The optimum conditions for biodegradations of pyrene, fluorene and fluoranthene were determined statistically using an experimental design of the L9 orthogonal array of the Taguchi method.^29^ The dominant optimum control factors for their corresponding biodegradation are as follows: for pyrene by Halobacillus B = 25°C, pH 7, 5% PAH concentration; for fluorene by Halomonas A = 40°C, pH 7, 1%, 3, 5% PAH concentration; and for fluoranthene by Halobacillus B = 25°C, pH 7, 5% PAH concentration.

### Polycyclic aromatic hydrocarbon degradation with optimum conditions

**Figure 4:**
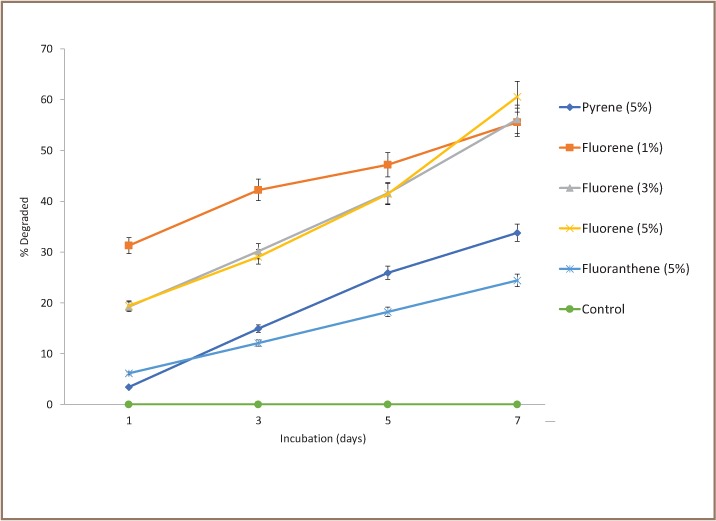
Time-course degradation of pyrene and fluoranthene by Halobacillus sp. B and fluorene by Halomonas sp. A under optimum conditions of temperature, pH, and PAH concentration defined by the Taguchi method (N = 3).

The assay was repeated to determine the extent to which the PAHs could be biodegraded by isolates Halobacillus B and Halomonas A with the application of the tripartite optimum control conditions of temperature, pH, and PAH concentration derived through the Taguchi method. Clear significantly increasing trends as a function of time were exhibited in the degradations of pyrene, fluorene and fluoranthene. The pattern of degradation fluorene > pyrene > fluoranthene was upheld (p<0.05), in particular after day 5 of incubation with Halobacillus B on high molecular weight (HMW) pyrene and fluoranthene and with Halomonas A on low molecular weight (LMW) fluorene as shown in Figure 4. Biomass yields of the degrader bacteria were also negatively correlated with degradations.

### Biodegradation by microbial co-culture

An assay was performed to test the hypothesis on the probable synergistic interaction between Halobacillus B and Halomonas A. Tests were performed as described in Marine Broth supplemented with 5% NaCl and with mixed cell suspension of both isolates in equal titer. The results showed that the two were indeed synergistic. Degradation after day 7 was up to 58.4% for pyrene, 63.7% for fluorene, and 44.8% for fluoranthene. Buildup of bacterial biomass and extent of PAH degradation were also negatively correlated.

### Test for enzymatic degradation on modified medium

A simple qualitative study was done to provide baseline information regarding the enzymatic degradation of the PAHs used. Profuse growth of both Halobacillus B and Halomonas A developed in media formulated with plain bacteriological agar (HiMedia, India), peptone and PAHs. Counterpart controls with the agar plus peptone and also only agar plus PAH had no growth or only nil to hazy growth.

## Discussion

The aim of the present study was to determine the PAH degradation capability of halophilic bacteria. It was found that the two isolates, Halobacillus B and Halomonas A were the best degraders of pyrene, fluorene and fluoranthene.

The concentrations used in the assay were low for pyrene and fluorene (0.08 mg/mL) and high for fluoranthene (0.4 mg/mL). Comparing pyrene (4-ring) and fluorene (3-ring), the degradation rate was faster with fluorene, but after 7 days this rate although changed a bit, did not differ significantly, reaching 3% compared to the degradation rate for pyrene using Gram-positive Halobacillus B, and not much compared with fluorene using Gram-negative Halomonas A. However, by the latter isolate alone, degradation was higher by 6% for fluorene (47%) over pyrene (41%). The implication of these comparisons is that HMW (4-ring) pyrene tends to be degraded better by Gram-positive and LMW (3-ring) fluorene better by Gram-negative degraders. A study by Zhou *et al.* showed six Gram-positive and five Gram-negative strains able to degrade HMW and LMW PAHs, respectively.^16^ Moreover, Halomonas species have genes that encode for ring-cleaving enzymes in the β-ketoadipate pathway for degradation of aromatic compounds. If by extrapolation the concentration of fluoranthene were reduced by dividing by 5 to equal that of fluorene, its breakdown by Halomonas A would be at least 17% lower in comparison to pyrene by the same isolate, and even much lower (37%) in comparison to fluorene by Halobacillus B.

In summary, the rate of degradation of a PAH is dependent on its molecular weight, number of benzene rings, volatility and solubility. Low molecular weight PAHs (with 2–3 rings) are relatively volatile and soluble in water, making them more degradable than others with HMW (with ≥4 rings).^30^ In this study fluorene, a LMW PAH, and pyrene and fluoranthene, both HMW, were degraded at significantly different rates by all 12 halophiles. Degradations by Halobacillus and Halomonas were 2–3 times higher compared to Chromohalobacter and Pontibacillus, indicating that potentials inherent in a genus and its species are also vital considerations in the biodegradation of these aromatic hydrocarbons. Growth rates of the degraders in PAH alone, especially those that form or do not form endospores, also need to be a foremost consideration. The low molecular weight PAH anthracene, for example, was utilized completely by the fast-grower alkaliphilic, Gram-positive Bacillus badius in as short as 60 hours.^30^

Size and its corollary, solubility in water, are likewise important, especially in the case of HMW PAHs such as in pyrene and fluoranthene. These determine the area of access for water and enzymes to leach in for both physical and biological degradation.^28^

Pyrene and fluoranthene are isomeric but vary in surface area (fluoranthene > pyrene, and solubility (pyrene = 0.14, fluoranthene = 0.26), making the former more readily degradable by all isolates tested in this study. However, culture conditions could lead to opposite results as in the study of Zhou *et al.* where fluoranthene was more degradable than pyrene.^16^

**Figure 5:**
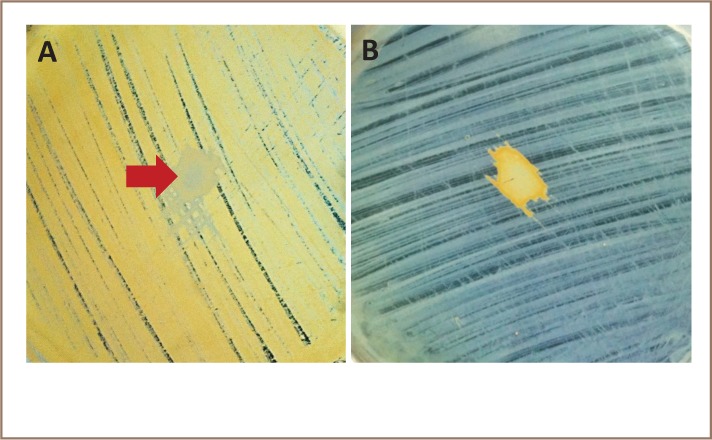
Test for reverse-antagonism-synergism of PAH degraders Halobacillus sp. B (HB) and Halomonas sp. A (HA). (A) HB, the “antagonized” with “antagonist” HA (Arrow). (B) HA and HB in reversed roles

Halobacillus B and Halomonas A, the two top PAH degraders, were brought together in reversed co-cultures on Marine Agar spiked with 0.05% NaCl (*Figure 5*). This was done to determine possible antagonism-synergism potentials between them. By chance, they differed in colony color, the former carotenoid-colored (orange) and the latter, pale white, thus, were easy to distinguish in the lawn spotting method of Long and Azam.^31^

No clearing zone developed up to 7 days to indicate antagonism by one isolate on the other in reversed co-cultures. On the other hand, that the two could be synergistic could only be hypothesized based on the findings by Oren.^32^ A large number of bacterial isolates (135) were garnered in this study from the two solar salt beds. ‘Helping-one-another’ is a widespread ecological phenomenon wherever populations in large communities coexist for continued survival in environments with finite resources. Colonies of Halobacillus B (CMS 1802), with its cells rich in carotenoid pigment, could provide a mantle of protection from solar radiation to colorless colonies underneath and in turn draw from the latter compounds for synthesis of its pigment. Previous studies have demonstrated that employment of microbial consortium results in more efficient biodegradation than one with pure culture. For example, Arulazhagan and Vasudevan obtained better degradation of PAHs in a marine environment with a consortium of moderately halophilic bacteria.^1^ However, the nature and behavior of the organisms involved and the level of contamination during biodegradation should be considered in such a complex synergistic or co-metabolic relationship.^27^

**Figure 6:**
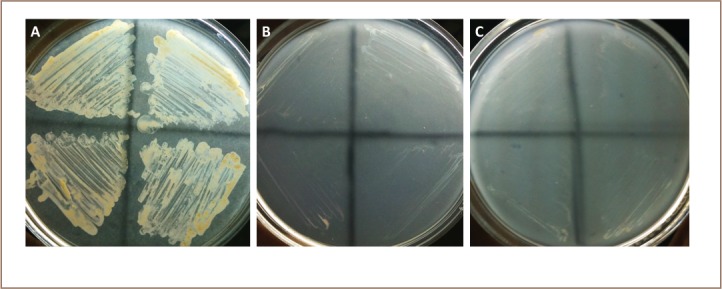
Qualitative test for enzymatic breakdown of PAH by Halobacillus sp. A. (A) profuse growth on plain agar + peptone + PAH. (B) hazy to nil growth on agar + peptone, (C) on agar + PAH

It was also determined that these two best degraders showed rich growth on the plain bacteriological agar, peptone and PAHs (*Figure 6*). Thus, other than by photooxidation, degradation of recalcitrant aromatics such as PAHs is enzymatic, most probably triggered by peptone, a major source of nitrogen used in microbiological culture media. Provision of N source to substrates does improve biodegradation via increase in biomass and accompanying synthesis of the four essential major biomolecules, including protein enzymes.^33^ The metabolic pathway in PAH degradation although still incomplete, is possible only with enzymes if the degrader microorganisms were to hydrolyze and use it as a carbon and energy source.

## Conclusions

Based on the results examined, halophilic bacteria showed a potential effect to tolerate PAHs at varying concentrations and degrade efficiently under optimal conditions. Moreover, genus Halobacillus and Halomonas have properties that are vital in biotechnological applications reducing aromatic hydrocarbons in natural environments.
